# The association between nutrition transition score and measures of obesity: results from a cross-sectional study among Latina/o immigrants in Baltimore

**DOI:** 10.1186/1744-8603-10-57

**Published:** 2014-07-07

**Authors:** Airín D Martínez, Hee-soon Juon, David M Levine, Victoria Lyford-Pike, Sadie Peters

**Affiliations:** 1 School of Transborder Studies, Arizona State University, P.O. Box 876303, Tempe, AZ 85287-6303, USA; 2 Department of Health, Behavior and Society, Johns Hopkins Bloomberg School of Public Health, 624 N Broadway, HH 7th Floor, Baltimore, MD 21205, USA; 3 Division of General Internal Medicine, Johns Hopkins School of Medicine, Johns Hopkins Outpatient Center, 601 N. Caroline Street, Baltimore, Maryland, 21287, USA; 4 The Arc of Baltimore, Inc., 7215 York Road, Baltimore, MD 21212-4499, USA

**Keywords:** Nutrition transition, Latino immigrants, Obesity, Self-perceived body weight

## Abstract

**Background:**

Studies suggest that US Latinos have a higher prevalence of obesity than White Americans. However, obesity may differ by pre-immigration factors and Latinos’ cultural representations of ideal body image. This paper explores whether country of origin’s stage in the nutrition transition is related to Latino immigrants’ BMI category and self-perception of weight.

**Methods:**

Primary data originated from a cross-sectional questionnaire of Latina/o immigrants in Baltimore in 2011. A convenience sample of self-identified Latinos, ≥18 years old, living in Baltimore was recruited from a community-based organization. Data for each country represented in the sample were obtained from the WHO Demographic and Health Surveys and the UN Food and Agriculture Organization. Each country was scored for its stage in the nutrition transition using a six-point scoring system. Descriptive statistics were conducted to characterize the sample. Bivariate analyses were conducted to examine the relationship between the outcome variables and the predictors. Multivariate logistic regressions were conducted to examine whether a country’s stage in the nutrition transition increased one’s odds of having an obese BMI score (≥30 kg/cm^2^) and perceiving one’s weight as overweight, while controlling for socio-demographic variables.

**Results:**

The sample (n = 149) consisted of immigrants from 12 Latin American countries. Participants lived in the US for x=10.24 years. About 40% of the sample had BMI ≥30 kg/m^2^ (obese). The longer Latina immigrants’ lived in the US, the less likely their country of origin’s nutrition transition score would increase their odds of having a BMI ≥30 kg/m^2^ (OR = 0.97 p < 0.04). The higher the country of origin’s nutrition transition score, the more likely BMI influenced Latino immigrants’ perception of their weight as above normal (OR = 1.06, p < 0.04). The effect of the nutrition transition score had a stronger effect on females than males.

**Conclusion:**

These results suggest that country of origin’s nutrition transition score and gender affect Latino immigrants’ objective and subjective measures of weight. Future investigation should investigate the relationship between gender and the nutrition transition in Latin America and how the nutrition transition globalizes obesity and weight consciousness.

## Background

Latino adults in the United States (US) have a higher prevalence of obesity than non-Hispanic Whites [1,2]. The relationship between extant acculturation and overweight/obesity among Latino immigrants has been well explored in the literature [3-10]. However, much less is known about the pre-immigration cultural and economic conditions that might predispose US Latino immigrants to becoming overweight/obese.

As is the case with many of the world’s nations, several Latin American countries have experienced a steady increase of overweight/obesity among its urban and rural populations [11,12]. For example, since the 1980s, Mexico has documented an increase in obesity in urban areas and continued malnutrition in the some rural areas [13]. This phenomenon, often labeled as “the nutrition transition” [13-16], is marked by the prevalence of overweight/obesity surpassing malnutrition and an increase in deaths from non-communicable chronic diseases (NCD) in low- and middle-income countries. The nutrition transition often precedes or occurs in tandem with demographic, epidemiological, and socioeconomic changes resulting from globalization, urbanization and development [17-19]. For example, Brazil, Chile, Ecuador, Mexico, Peru, and the Dominican Republic are considered to be advancing in the nutrition transition because since 2000 these countries have had an increasing prevalence of obesity, increased proportion of dietary intake from fat, reducing prevalence of infant mortality and stunting, and increasing NCD mortality [20-28].

It is not known whether Latino immigrants from countries in the receding famine (Stage 3) or nutrition-related non-communicable disease stages (Stage 4) of the nutrition transition perceive overweight/obese as normal, more so than those from less than developed Latin American countries. It is possible that the normative acceptance of fuller bodies might be attributed to a physical acceptance of the widespread presence of obesity or its association with wealth and status. In the US, researchers have found that racial and ethnic minorities often misperceive their overweight/obesity and do not attempt weight loss [29]. This paper sets out to explore whether country of origin and its stage in the nutrition transition affects Latino immigrants’ objective and subjective measures of overweight/obesity. Our hypotheses were 1) overweight/obese Latinos will perceive their weight as less than normal/normal; 2) country of origin’s stage in the nutrition transition score will positively increase the odds of Latino immigrants belonging to the obese body mass index (BMI) category (BMI ≥30 kg/m^2^); and 3) country of origin’s nutrition transition score will positively increase the odds of overweight/obese Latino immigrants to perceive their weight as underweight or normal.

## Methods

The current study utilized both primary and secondary data to analyze how country of origin’s nutrition transition affects BMI and self-perception of body weight within a sample of Latino immigrants in Baltimore. Primary data were collected from a community-based participatory cardiovascular risk assessment of Latina/o immigrants in Baltimore in 2011. This study was a single-step, cross-sectional questionnaire with objectives to examine cardiovascular risk factors, knowledge, attitudes and behaviors of Baltimore Latinos.

Participants were recruited as a convenience sample from a pool of clients utilizing the partnering community-based organization for health, social, legal, and educational services. Participants were enrolled if they self-identified as Latina/o, were at least 18 years of age, and had a zip code within the limits of the Baltimore Metropolitan area. The survey was administered in a face-to-face format to circumvent potential low levels of literacy among participants. The instrument included a food security scale [30] and was piloted with 10 clients at the participating community-based organization. After pilot participants reported respondent fatigue to the original 90-minute session, all scales were replaced with existing shorter versions and the instrument was carefully parsed to omit all redundant phrases. All interviews were conducted in the participant’s language of choice, and all participants chose to conduct their interview in Spanish. Interviews lasted approximately sixty minutes. The Johns Hopkins School of Medicine Institutional Review Board approved this study.

We obtained secondary data about the nutrition status in the participants’ country of origin from the WHO Global Health Observatory Database [31] and the UN Food and Agricultural Organization of the United States (FAO) Food Balance Sheets [32].

### Outcome measures: objective and subjective measures of overweight and obesity

In our study, body weight was measured both objectively by measuring weight and height and calculating BMI, as well as subjectively by querying self-perception of body weight. A trained research assistant weighed participants and measured their height on a Detecto 439 Mechanical Doctor Scale with height rod. The staff at St. Joseph’s Hospital in Baltimore calibrated the scale prior to use. The body mass index (BMI = kg/m^2^) is a proxy for human body mass that is often used in national and international datasets. BMI between 18.5-24.9 kg/m^2^ is considered normal. BMI between 25–29.9 kg/m^2^ is overweight, and at or above 30 kg/m^2^ is obese [33]. Self-perception of body weight was assessed with a question from Muñoz and colleagues’ [34] survey: “Do you think your weight is a) less than normal, b) normal, or c) more than normal?” We adapted the self-perception of body weight into a binary outcome variable to conduct logistic regression. Participants who assessed their weight as “less than normal” and “normal” were coded as 0, while those who perceived their weight as “above normal” were coded as 1.

#### Food insecurity

In diverse populations in both developed and developing nations, studies have found a mild to strong relationship between food insecurity and obesity [35,36]. Food insecurity is a state in which one’s availability of nutritionally adequate foods, or one’s ability to acquire acceptable food, is limited or uncertain. Food insecurity was used to discern material access to food and hunger. Food insecurity was measured using the Short Form of the Food Security Survey Module [30], which has demonstrated validity in other continents. Food security was scored using Bickel and colleagues’ guidelines [37], in which affirmative items are scored with “1”. The scale is from 0–7, in which zero represents households that are food secure with no risk, while a cumulative score above 4 represented households that are food insecure with moderate hunger. The inter-item reliability for this scale was α = 0.87.

### Nutrition transition score

To investigate the factors related to overweight and obesity among Latina/o immigrants, it is important to consider the context of the participants’ country of origin, as weight gain may not simply be associated with the attainment of deleterious, Western dietary practices in the US, but could also result from conditions in their sender country [38-40]. For this reason, we included a measure for the stage in the nutrition transition for the participants’ country of origin. For each country, we examined percentage of the population living on < $1/day, infant mortality rate (IMR), NCD mortality percentage (both cancer- and chronic disease-related deaths), and the prevalence of BMI in the population in 2008 from the WHO Global Health Observatory Database. We obtained data regarding total energy intake and percentage of energy intake from fat from the FAO Food Balance Sheets for each country.

With these six indicators we were able to score each of the countries represented in the sample for their stage in the nutrition transition, as described by Abrahams, Mchiza and Steyn [20]. This six-point scoring system locates each country within one of these stages of the nutrition transition by assessing both epidemiological and socio-demographic indicators. We gave countries one point if they were in the top quartile for total energy (kcal per day), percentage of energy from fat (fat kcal per day/total kcal per day), overweight/obesity prevalence (≥25 kg/m^2^), and NCD percentage mortality rates. Countries that were at the bottom quartiles were given a “0.” Alternatively, countries were allotted one point if they were in the bottom quartile range for percentage of people living with < $1/day and the prevalence of IMR. The points were then added for each country. We did not calculate the quartiles for this scoring system using all 21 Spanish-speaking Latin American countries only those represented in the sample because the differences between countries would be much greater (See Table 1).

**Table 1:** Latin American countries listed in descending order of Nutrition Transition Scores and the indicators that contribute to them

**Country**	**PPP**	**PPP Score**	**Total kcal**	**Total kcal score**	**% Energy from fat**	**% Energy fat-score**	**IMR**^**1**^	**IMR Score**	**NCD Mortality %**	**NCD Score**	**Overweight/Obese %**	**OV/OB score**	**Total score**
Colombia	11.32		2707		24.2%		19		66		48.3		0
El Salvador	8.97		2587		20.2%		20		67		61.1		0
Guatemala	13.53		2226		23.7%		33		47		51.5		0
Honduras	21.36		2687		24.7%		25		69		50.1		0
Dominican Republic	3.30		2451		30.8%		28		68		54.3		0
Peru	6.20		2520		15.2%		22		59		46.3		0
Ecuador	6.45		2271		36.3%	1	24		65		55		1
Brazil	6.14		3171	1	31.5%	1	21		74		51.7		2
Venezuela	6.63		2783		26.3%		16	1	66		66.9	1	2
Costa Rica	2.42	1	2876		27.6%		9	1	80	1	58.3		3
Mexico	1.15	1	3188	1	26.7%		17		78	1	68.3	1	4
Argentina	1.94	1	2974	1	33.1%	1	15	1	80	1	64.2	1	6

PPP-percentage of the population living < $1/day.

IMR-infant mortality rate.

NCD-non-communicable diseases.

OV/OB-overweight/obese.

1. All data from 2008, except IMR is from 2009.

There are five stages in the nutrition transition: 1) hunter-gatherer or Paleolithic; 2) modern agriculture and famine; 3) receding famine (as incomes grow); 4) degenerative disease, in which changes in activity levels and diet lead to increased levels of non communicable diseases (NCDs); and 5) behavioral change in which populations reduce their fat, increase fiber intake, and do meaningful physical activity that extends mortality and reduces NCD [17]. Therefore, a score of 0 in the six-point scale demonstrated a country being closer to the Paleolithic stage, while a score of above 5 represented the behavioral change stage.

#### Demographics

Our main demographic variables are age, education level, gender, and time in US. Time in US was included as a proxy for acculturation. Given that more recent arrivals are more likely to be affected by the conditions in their country of origin, we also created two variables to analyze those immigrants living in the US less five years and more than 10 years. Age and time in US were continuous variables, while gender was a dichotomous variable.

#### Statistical analyses

Descriptive statistics were conducted to describe the sample’s demographic characteristics. To examine hypothesis #1, whether Latinos with higher BMI scores will perceive their weight as normal or underweight, we did a chi-square analysis between BMI categories (non-obese and obese) and self-perceived body weight (less than normal/normal and above normal). In addition, a one-way ANOVA was calculated to examine whether there was a difference in the means of BMI scores by self-perception of body weight. We calculated correlations between the predictor and outcome variables to identify whether the relationship between the variables was statistically significant and whether the association was positive or negative. Spearman rank correlations were used to calculate the relationship between categorical predictor variables such as gender, education level, food security, and geographic region.

To examine hypothesis #2: country of origin’s nutrition transition score will positively increase the odds of Latino immigrants belonging to the obese BMI category, we conducted a multivariate logistic regression as we divided BMI scores into non-obese (≤29.9 kg/m^2^) and obese (≥30 kg/m^2^) categories. Similarly, for hypothesis #3: country of origin’s nutrition transition score will positively increase the odds of Latino immigrants perceiving their weight as less than normal/normal, we did the same as it was a dichotomous outcome. All regressions were adjusted for age, education level, food security, gender, and time in USA. All analyses were performed with STATA Version 12.

## Results and discussion

### Description of the sample and countries of origin

The sample was a relatively low-educated sample of immigrants representing 12 countries in Latin America and Puerto Rico. Most of the sample consisted of single (~55%) females (76%) with mean age of 38 years. Over half of sample originated from a Central American country. Approximately 21% of the sample had moderate food insecurity, while 19.5% were food insecure with some hunger at the household level. Over 72% of the sample was overweight/obese (See Table 2).

**Table 2:** Sample demographic characteristics

	**Total (*n =*** **149)**
**Mean age** ± SD, range	38.95 ± 11.48, 20-77
**Years in the US** ± SD	10.24 ± 10.12
**Years in Baltimore** ± SD	7.56 ± 9.09
**Gender**	
Female	(98) 65.7%
Male	(51) 34.3%
**Latin American Region**	
Mexican	(38) 25.5%
Central American	(87) 58.4%
South American	(24) 16.1%
**Education (*n*** **= 146)**	
None	(8) 5.48%
Less than 8 years	(80) 54.79%
Some High School	(19) 13.01%
High School Graduate	(15) 10.27%
Some College	(16) 10.96%
Completed college or	
higher	(8) 5.48%
**Food security**	
No food insecurity	(74) 49.66%
Some risk for food	
insecurity	(15) 10.07%
Moderate food insecurity at the household level	(31) 20.81%
Food insecure with some hunger at the household level	(29) 19.46%
**BMI** <30 kg/m^2^	(89) 59.73%
**BMI** >30 kg/m^2^	(60) 40.27%

The scores for the country of origin’s stage in the nutrition transition are shown in Table 1. We were able to obtain all of the data necessary to complete the nutrition transition six-point scoring system [20] for all 12 countries represented in the sample for the year 2008. Puerto Rico was excluded from the scoring because it is a territory of the United States and not part of the WHO Global Health Observatory or the FAO Food Balance Sheets.

The countries represented in our sample vary in their stage in the nutrition transition. Guatemala was in the lowest quartiles for all of the six indicators except increasing prevalence of overweight/obesity. In contrast, Argentina and Mexico were furthest along in the nutrition transition, with Argentina having a perfect score (6). Regardless of the nutrition transition score, the prevalence of overweight/obesity and the prevalence of NCD mortality were close to or exceeded 50% in each country.

#### Exploratory data analysis

Although BMI is a continuous variable it was not normally distributed because of the high prevalence of overweight/obesity in this sample. To conduct our bivariate analysis and regression analyses, we converted the BMI scores to a dichotomous variable, non-obese (≤29.9 kg/m^2^) or obese (≥30 kg/m^2^). Previous publications split their samples’ BMI scores using this criterion [28]. To conduct the one-way ANOVA examining whether there was a difference in the means of BMI scores by self-perception of body weight, the BMI scores were logarithmically transformed.

The results of the correlations between the independent variables and the predictor variables can be found on Table 3. Age (r = 0.17, p < 0.05) was positively correlated with binary BMI categories, while gender (1 = F, 2 = M) was negatively correlated with BMI categories (r = -0.22, p < 0.001) and self-perception of body weight (r = -0.22, p < 0.01), suggesting a stronger relationship with females. Food security also had a negative relationship with self-perception of body weight (r = -0.22, p < 0.01), suggesting that more food security was strongly related to perceiving one’s weight as above normal (See Table 3). Consequently, we added age, gender, food security and time in the US in the logistic regression models to see if the effect of nutrition transition score on our outcome variables remained significant. Education level and geographic region were included in the exploratory regression models to examine whether they would influence the other predictors.

**Table 3:** Correlations between outcome variables and predictor variables

	**Binary BMI categories**		**Self-perceived weight**	
	**r**	**p-value**	**r**	**p-value**
**Age**	0.17	0.05*	0.07	0.40
**Gender**^**§**^	-0.22	0.001***	-0.22	0.01**
**Education**^**§**^	-0.01	0.94	0.04	0.63
**Food Security**^**§**^	-0.11	0.19	-0.22	0.01**
**Geographic Region**^**§**^	0.04	0.63	0.07	0.38
**Nutrition Transition Score**	0.69	0.40	0.03	0.70
**Years in the US**	0.12	0.28	0.16	0.05*
**More than 10 years in US**	0.27	0.08	0.14	0.10

^§^Spearman rank correlation calculated.

*p < 0.05, **p < 0.01, ***p < 0.001.

#### The relationship between objective and subjective measures of obesity

In a chi-square analysis, binary BMI categories and self-perception of body were strongly related (χ^2^ = 23.39, p < 0.001). We continued our analysis with a one-way ANOVA to calculate the difference in the means of the log BMI scores by self-perception of weight categories (less than normal/normal and above normal). The mean log BMI scores differ significantly (F = 20.82 p < 0.001) between those who perceive their weight as less than normal/normal and those who perceive their weight as above normal. Persons who perceive their weight as above normal have higher mean log BMI scores (× = 3.43) than those persons who perceive their weight as less than normal/normal (× = 3.28). Therefore, we had to reject our hypothesis that Latino immigrants would perceive their overweight/obesity as less than normal/normal.

#### The effect of nutrition transition score on objective and subjective measures of weight

The results from the multivariate logistic regression examining BMI category demonstrated that the country of origin’s stage in the nutrition transition increases the odds of Latina/o immigrants being in the obese BMI category (OR = 1.48, p < 0.01), the older they were (OR = 1.04, p < 0.06). However, given that gender was so strongly related to BMI category in our bivariate analysis and that women made up 65.7% of the sample, we also calculated the logistic regression models by splitting the sample by gender. These results reveal that indeed country of origin’s nutrition transition score increases the odds of being in the obese BMI category more in females (OR = 1.825, p < 0.01) than in males. Nevertheless, given the number of males (n = 49), the effect of nutrition transition score remains positive, but insignificant (OR = 1.136, p < 0.71).

**Figure 1:**
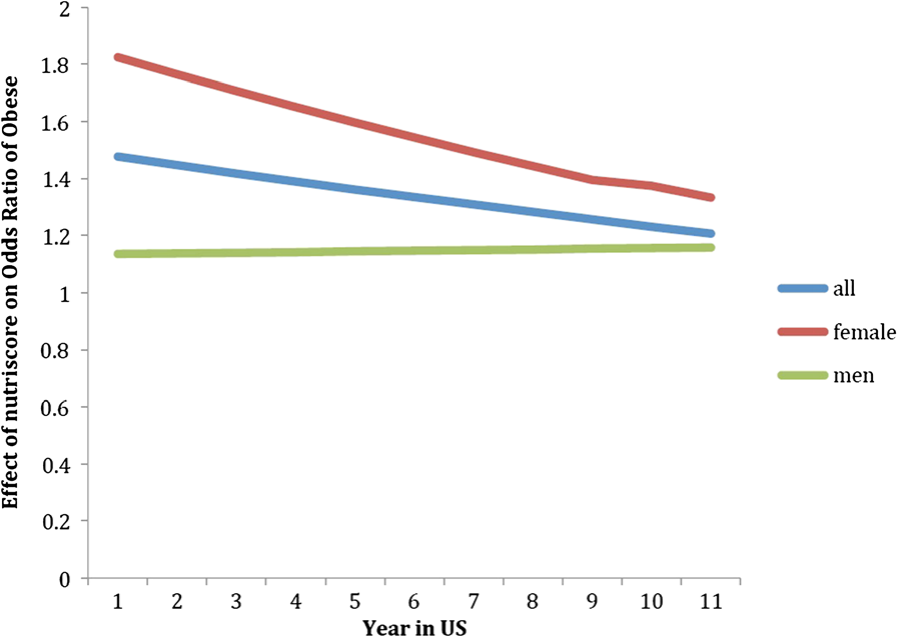
The interaction effect of nutrition transition score and years in US on BMI obese category.

Taking into consideration that the longer immigrants live in the US, the less their country of origin will affect their weight, we conducted a logistic regression of BMI categories with an interaction between time in the US and country of origin’s nutrition transition score. Here, we hypothesized that the longer Latina/o immigrants lived in the US, the less their country of origin’s nutrition transition score will affect their odds of being in the obese BMI category. There was a negative, yet significant effect of this interaction term on BMI category. Latina/o immigrants’ country of origin’s nutrition transition score will be less likely to increase their odds of being in the obese BMI category the longer they live in the US (OR = 0.978, p < 0.01) (See Figure 1, Table 4, Column 1). These results remain constant after inserting age, gender, food security, education and self-perception of body weight. We also calculated this logistic regression model by gender and found that country of origin’s nutrition transition score (OR = 1.825, p < 0.01) and the interaction term between time in the US and nutrition transition score is mainly significant among females (OR = 0.967, p < 0.05), and not males (See Figure 1).

**Table 4:** Results of multivariate logistic regressions of BMI categories and self-perception of weight

	**1. Binary BMI**^♦^	**2. Female binary BMI**	**3. Self-perception of weight**^♦^	**4. Self-perception of weight**^♦^
	**(non-obese/obese)**	**(non-obese/obese)**		
**Independent variables**				
**Age**	1.037	1.069	0.988	0.988
	(0.020)*	(0.029)**	(0.019)	(0.019)
**BMI**			1.105	1.067
			(0.070)	(0.063)
**Education level**	0.939	0.788	1.057	1.071
	(0.192)	(0.202)	(0.200)	(0.213)
**Food security**	0.986	1.008	0.693	0.709
	(0.170)	(0.214)	(0.100)***	(0.105)**
**Gender**	0.607		0.476	0.463
	(0.270)		(0.194)	(0.189)*
**Nutrition transition score**	1.476	1.825	1.018	0.158
	(0.206)***	(0.426)***	(.100)	(0.137)**
**Self-perception**	6.332	7.634		
	(2.692)***	(4.227)***		
**Years in US**	1.033	1.030	0.991	1.001
	(0.027)	(0.030)	(0.019)	(0.021)
**Nutrition transition score**^**##**^**BMI**				1.067
				(0.032)**
**Years in US**^**##**^**nutrition transition score**	0.978	0.967		
	(0.007)**	(0.015)**		
**N**	145	96	144	144

^♦^Robust standard errors.

p < 0.10*, p < 0.05**, p < 0.01***.

^##^Interaction.

**Figure 2:**
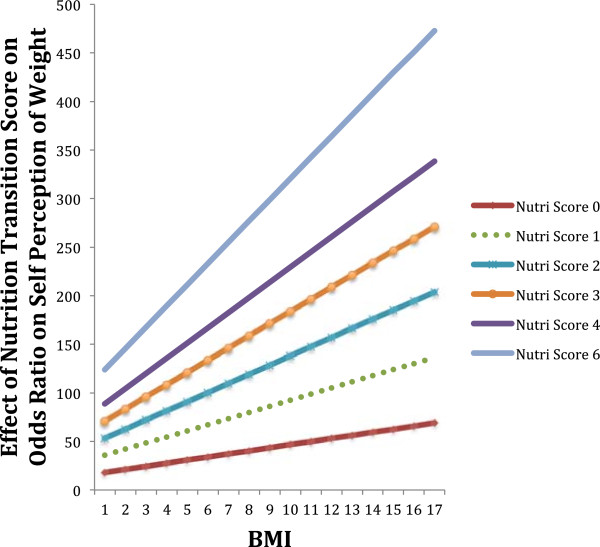
The interaction effect of nutrition transition score and BMI score on self-perception of body weight.

In testing our final hypothesis, that the country of origin’s stage in the nutrition transition directly decreases the odds of Latina/o immigrants perceiving their weight as above normal after inserting age, gender, education, food security, and time in US into the model, we did not find significant results for the main effect. Considering that this sample’s objective and subjective measures of obesity were so strongly related, we explored whether Latina/o immigrants with higher BMI scores were more likely to perceive their weight as above normal in an interaction with nutrition transition score. The hypothesis here was that country of origin’s nutrition transition score would increase the odds of perceiving one’s overweight as above normal given their actual weight. In this logistic regression model with nutrition transition score in an interaction with BMI score, we found that Latina/o immigrants from countries with a higher nutrition transition score have greater odds of perceiving their high BMI as above normal (OR = 1.07, p < 0.05). These results remained significant after inserting age, gender, education, food security, and time in US in the logistic regression model. Length of time in the US alone does not seem to affect Latina/o immigrants’ odds of being in the obese BMI category or one’s self-perception of body weight, except for BMI category in an interaction with nutrition transition score (See Table 4, Column 4). The country of origin’s stage in the nutrition transition seems to influence how one interprets their objective weight (See Figure 2).

## Conclusion

Given the increasing prevalence of obesity in Latin American countries [11-13,22-28], we commenced this exploratory study attempting to discern whether country of origin’s nutrition transition score, as a proxy for demographic, epidemiological and nutritional development, would effect Latino immigrants’ objective and subjective measures of obesity. The nutrition transition represents a society’s transition in dietary consumption and physical activity that results from globalization, epidemiological and demographic transitions [14,17]. Globalization accelerates the nutrition transition by modernizing food systems in low- to middle-income countries and by making energy-dense, nutrient deficient processed foods and meat more readily available. These specific foods increase total caloric intake and proportion of energy from saturated and hydrogenated fats [17]. The demographic shift represents the increasing urbanization and the subsequent lifestyle changes that require less energy expenditure from improved transportation and less physically taxing labor, among other factors [16,18]. The epidemiological shift represents an increased prevalence of non-communicable and degenerative diseases in populations that had higher levels of infant mortality and lower life expectancy. The increase in overweight/obesity, non-communicable and degenerative diseases are due to improvements in nutritional deficiencies and technological advancements [14,17,19].

This was largely a female and moderately food insecure sample. Most of the sample had lived in the US anywhere from a few months to 45 years, with an average of 10 years. Latino immigrants in this sample represented 12 Latin American countries. The diversity of Latino subgroups in this sample is often not found in many areas of the US often having a high concentration of Latinos from two or three countries. We found that this sample of Latino immigrants generally came from obesogenic countries. Argentina was at Behavioral Stage 5, which is not surprising because they are the third largest economy in Latin America. Guatemala was in the lowest quartiles for demographic, epidemiological and nutritional development at zero. Nonetheless, this is not to say that aspects of development do not exist within pockets of a country’s metropolitan elite and higher strata.

For our first hypothesis, we found that persons with higher BMI scores often did perceive their weight as above normal. This contradicted our initial assumption based on the extant literature that Latinos with overweight/obesity do not believe that they need to change their dietary behaviors [29] and that Latinas find fuller bodies more attractive or symbolic of wealth and status [41-49]. Not only were these participants from obesogenic countries, but they themselves perceived their overweight/obesity as above normal. The objective measures of obesity matched Latina/o immigrants’ subjective perceptions of obesity.

For our second hypothesis, Latino immigrants’ country of origin’s stage in the nutrition transition positively increased the odds of being in the obese BMI category. When we split the sample by gender, this effect was stronger among women. Previous research has demonstrated that Latinas have a higher propensity to maintain weight gain after childbearing [47]. In addition, Latina immigrants do not engage in the same physically demanding labor as Latino immigrants, preventing adequate energy expenditure in their host society [48].

Considering that one’s country of origin will affect their weight less the more time they live as immigrants, we included an interaction term between time in US and nutrition transition score. The effect of the nutrition transition score on BMI category faded the longer Latina immigrants lived in the US. We estimated that by year 19–20 of living in the US (x = 19, y = 1.005) the nutrition transition score would have no effect on BMI. So, for women who arrived before the early 1990s, the nutrition transition of their respective country affected their objective measures of obesity in the US less. This resonates with periods of development in Mexico with the enactment of the North American Free Trade Agreement in 1994, and with our South American countries’ convergence in the South American free trade agreement, MERCOSUR, in 1991.

Leaving an obesogenic environment and entering another may perpetuate poor dietary practices and sedentary lifestyles, albeit at different lengths of time. These results also motivate us to further identify those aspects of Latina/o immigrants’ diets that were Western prior to arriving to the US and their physical activity. This could help researchers in the field better understand the conditions from the host society that precisely change health behaviors.

We also entered this exploratory study assuming that immigrants from countries in the lower stages of the nutrition transition, specifically Stages 2 (Modern Agriculture and Famine) and Stage 3 (Receding Famine), would be less likely to perceive their higher weight as above normal, given the literature of fuller figures being symbolic of status and wealth in developing countries [49]. We discovered that the more advanced one’s country of origin was on the nutrition transition trajectory the more likely one’s objective measure of obesity corresponded to their subjective measure of obesity.

This examination reveals that self-perception of body weight is not exclusively related to nutrition transition score. Behavioral Stage 5 of the nutrition transition is supposed to be the stage in which the society realizes their energy-dense diets and sedentary lifestyles are problematic given increasing non-communicable disease mortality rates and reduced quality of life from degenerative diseases. However, most of the participants came from countries low in the nutrition transition trajectory (n = 107), with a score of zero or one, and they perceived their weight as above normal. There are processes at play shaping Latina/o immigrants’ norms and beliefs of body image.

For instance, those with more food security in this sample had higher odds of perceiving their weight as above normal. People with more food security may have more economic resources and thinner ideals of body image may be related to improved socioeconomic status. In a sample of Colombian women of low- and middle-income, Gilbert-Diamond and colleagues [50] found that women with more household assets found thinner body shapes to be more ideal. Future research should examine how improved food security changes body image ideals and Latina/o immigrants’ self-perception of body weight.

One of the factors contributing to the nutrition transition is globalization, particularly the increased local production, marketing, distribution and consumption of processed foods. Yet, parallel to the growth of the advertising of processed foods in low- and middle-income countries, is the global dissemination of weight-conscious discourses from public health or advertising. For example, in ten different countries, Brewis and colleagues [47] found that fat-ness is becoming stigmatized as both socially unacceptable and unhealthy at a striking pace. Their Mexican and Paraguayan participants had the highest fat stigma scores, in a sample that included participants from developed countries such as the US and UK.

Alternatively, beliefs and norms of ideal body images may be related to current norms in the US. For example, Vladrich and colleagues [43] found that Latinas’ individual psychosocial measures demonstrated a preference for thinner bodies, but in the presence of other Latinas in a focus group, they embraced curvy body shapes as a counter image to thin ideals in the US. Unfortunately, our original survey did not include questions about the participants’ general perceptions of body shape or obesity. In the future we plan to measure Latina/o immigrants’ general perceptions of body shape and obesity to better understand how different people perceive their weight in comparison to others.

The implications of these findings for obesity research with Latino immigrants in Western countries, particularly the US, is that it challenges the assumption that obesity is a direct result of factors in host society, or acculturation. In the bivariate analyses and the regression models, time in US did not influence BMI scores or self-perception of body weight. However, time in US was still important in understanding how immigrants’ country of origin continues to affect their BMI category. As our results suggest, Latino immigrants could have been overweight/obese in their country of origin. It would be important to query in future research if people were overweight in their country of origin and whether a doctor in their country of origin ever told them that they needed to lose weight. New environmental, structural, and cultural conditions in a more developed host society can affect overweight/obesity among Latino immigrants, but the critical catalytic or facilitating factors remain somewhat obscure.

### Limitations

There was a high prevalence of overweight and obesity in this sample of Latina/o immigrants, so we did not have a normal distribution for BMI scores. To resolve this issue, we transformed the BMI scores to log BMI scores for the one-way ANOVA and created a binary BMI outcome variable for the multivariate logistic regression. This may have been a result of selection bias, since we recruited among Latinos actively seeking services at a community-based organization in East Baltimore. It is also the result of having a smaller sample size. This sample may not be representative of the foreign-born Latinas/os in Baltimore, but this is a hard-to-reach population given the chilling effect of immigration enforcement policies that were in place during the time of the data collection (i.e., Secure Communities). Moreover, there is a limited amount of health data available about the Hispanic/Latino ethnic group living in Baltimore, Maryland and we are providing preliminary results that will surely help our future goals to conduct a larger study with a larger population and a refined instrument.

The nutrition transition score that we calculated is based on data available from the WHO Global Health Observatory Database for 2008. The availability of data prior to the year 2008 for PPP, NCD mortality rates and percentage of the population with overweight/obesity prevents us from creating a nutrition transition score for each country that is aligned with our participants’ year of arrival to the US. Therefore, the nutrition transition score although helpful in allowing us to capture Latina/o immigrants’ country of origin’s economic development, is incomplete. We tried to ameliorate this limitation by including an interaction term between time in US and nutrition transition score. There is a need to continue this investigation in the future as more data become available.

Regions within the same country can be in different stages of the nutrition transition. The nutrition transition, as with any indicator of development, does not equally affect persons in rural or isolated regions. The data collected in the CVD risk assessment did not query the participants’ region within their country of origin, but this should be considered for future research. Having these details may help elucidate variance among immigrants from the same country. More populated and developed urban metropolises may skew the data for the entire country. Areas for further investigation include a follow-up study examining how nutrition transition affects BMI scores with a larger sample of Latino immigrants, in other cities or immigrant destinations, or a secondary data analysis of a dataset that has a diverse representation of Latinos immigrants as this one.

There are also limitations with some of the measures that we used. For example, BMI scores do not take into consideration lean muscle mass, the proportion of body fat to muscle, or the waist-to-hip circumference ratio. We would have had a more comprehensive set of objective measures of weight if we included these measures in the study. Our use of the Short Form Food Insecurity Scale [30] does not measure the most severe form of food insecurity, which is child food insecurity and hunger. Short forms of all the scales were utilized in the original questionnaire because of the original breadth and aims of the survey, and to avoid respondent burden.

Adding country of origin’s nutrition transition score to our regression models examining objective and subjective measures of obesity among Latino immigrants in Baltimore, was an attempt to acknowledge Latino immigrants’ heterogeneity and their pre-immigration factors. Although many examining the changing perceptions of body image in developing countries acknowledge the nutrition transition as a possible explanation for the growing global prevalence of obesity in low- to middle-income countries, very few [38] integrate this construct into their analysis of self-perception of body weight. We contribute to this discussion on the changing norms around ideal body image and obesity by demonstrating in a modest way that nutrition transition is one of the processes related to Latina/o immigrants’ BMI and self-perception of body weight.

## Competing interests

The authors declare that they have no competing interests.

## Authors’ contributions

ADM and HJ conceived the study concept and design for this study. The parent study was conceived and designed by SP. ADM, VLP, and SP acquired the primary data. ADM acquired the secondary data. ADM and HJ statistically analyzed the data and interpreted the results, while DL, VLP, and SP also interpreted the results. ADM, HJ, DL, VLP, and SP made critical revisions of the manuscript for important intellectual content*.* SP supervised the overall study. All authors read and approved the final manuscript.
